# Automated speech-fluency explanations for schizophrenia diagnosis

**DOI:** 10.1038/s41598-025-33129-w

**Published:** 2025-12-22

**Authors:** Rok Rajher, Mila Marinković, Polona Rus Prelog, Jure Žabkar

**Affiliations:** 1 Faculty of Computer and Information Science, University of Ljubljana, Večna pot 113, 1000 Ljubljana, Slovenia; 2 Centre for Clinical Psychiatry, University Psychiatric Clinic Ljubljana, Chengdujska 45, 1260 Ljubljana, Slovenia; 3 Faculty of Medicine, University of Ljubljana, Vrazov trg 2, 1000 Ljubljana, Slovenia

**Keywords:** Automated schizophrenia detection, Automated speech recognition, Verbal fluency, Computational biology and bioinformatics, Diseases, Health care, Mathematics and computing, Medical research

## Abstract

Schizophrenia is a chronic and severe mental disorder that still relies on time-intensive, clinician-administered assessments. Although several automated approaches have been proposed to support diagnosis, these systems often lack the level of explainability necessary for informed clinical decision-making. In this study, we present a fully automated and explainable pipeline for detecting schizophrenia from audio recordings of verbal fluency tests, collected from 126 Slovene-speaking participants (68 healthy controls, 58 individuals diagnosed with schizophrenia), leveraging recent advancements in automatic speech recognition (ASR) and large language model (LLM) systems. We evaluated three ASR models–Truebar, Whisper, and Soniox–for transcription quality, and selected the best-performing system for further processing. We semantically enriched the transcriptions using the generative capabilities of LLMs and extracted both verbal and non-verbal features grounded in established diagnostic criteria. We assessed the relevance of these features using a Bayesian statistical framework and trained multiple classical machine learning models for automatic classification. Our best-performing model, an Explainable Boosting Machine, achieved a classification accuracy of 0.82 and an AUC of 0.90. We further generated visual explanations for the model’s predictions, establishing the first fully automated and explainable schizophrenia detection framework developed for the Slovene language. Our approach prioritizes explainability through model-transparent outputs, while still achieving performance comparable to existing automated systems for speech-based schizophrenia detection.

## Introduction

Schizophrenia is a chronic and severe mental disorder^1,2^ that affects how an individual thinks, feels, and behaves. It is classified as a psychotic disorder and is characterized by a combination of hallucinations, delusions, disorganized thinking and behavior, and emotional blunting^3,4^. These symptoms often cause significant disruption to an individual’s social, academic, and occupational functioning and, in severe cases, can lead to prolonged hospitalization and lifelong care^5,6^. Based on Global Burden of Disease 2019 estimates, the global age-standardized point prevalence of schizophrenia in 2019 was 287 per 100,000 (0.287%)^7^ while according to WHO^8^, the global prevalence of schizophrenia is estimated at approximately 0.29%, with around 23 million people affected worldwide as of 2025.

Currently, there is no objective or standardized diagnostic test for schizophrenia. The most widely used diagnostic frameworks in clinical practice are the DSM-5^3^ and ICD-11^4^. Both are clinician-administered classification manuals that provide formal diagnostic criteria and guide clinicians in assessing symptoms based on observed behavior, self-reports, medical history, and mental state.

While these diagnostic protocols are well-established, they are inherently subjective, time-consuming, and dependent on the clinician’s experience and expertise. With recent advances in automatic speech recognition (ASR) and large language models (LLMs), there is growing interest in developing computational tools that can support, augment, or partially automate aspects of psychiatric assessment. Such tools could serve as clinical decision-support systems, offering consistency, scalability, and assistive reasoning during the diagnostic process.

In this study, we focus on the verbal fluency task (VFT), which is a widely used test in neuropsychological assessment. In a verbal fluency task, participants rapidly generate words under constrained rules for a fixed interval (typically 60s). The most common variants are semantic/category fluency (e.g., “animals”) and phonemic/letter fluency (e.g., words beginning with “L”). The performance reflects lexical access, executive control (clustering and switching), processing speed, working memory, and semantic memory, and is frequently impaired in schizophrenia. Within VFTs, two error types–intrusions and neologisms—are particularly informative for disorganization. Intrusions are responses that violate the task constraint (e.g., off-category items during semantic fluency: “table” for animals) or words that do not begin with the target letter during phonemic fluency (e.g., “river” for letter “L”). Neologisms are newly constructed words that cannot be found in a lexicon (e.g., “lioner” or “animalling”) and reflect abnormal word formation. We find VFT well-suited for automation: ASR can produce transcripts, LLMs can do post-editing and semantic tagging, and interpretable ML models can support decision-making in clinical practice.

Previous research has demonstrated the potential of automatic speech-based analysis and ASR systems for schizophrenia classification. Chakraborty et al.^9^ used low-level acoustic features to predict negative symptoms in English-speaking patients, achieving accuracies between 79% and 86%. Xu et al.^10^ applied automatic transcription to clinical interviews conducted in English and extracted a combination of verbal and non-verbal features (phonatory, prosodic, articulatory, and conversational), achieving classification accuracies between 69% and 75%. In a follow-up study, Xu et al.^11^ integrated semantic coherence measures extracted from ASR transcripts into their multimodal audio–visual model, further improving classification accuracy to 82%. Ciampelli et al.^12^ combined ASR-generated transcripts with semantic natural language processing models to classify schizophrenia in Dutch-speaking participants, reaching accuracy of 77%. Although these pipelines are fully automated, they compromise explainability, either through the use of opaque feature representations or inherently unexplainable model architectures.

Beyond acoustic modeling, natural language processing (NLP) and machine learning (ML) methods have been increasingly applied to analyze linguistic and semantic abnormalities in schizophrenia. Early computational studies examined lexical diversity, coherence, and syntactic structure to quantify disorganized speech^13–15^. Graph-based and embedding-based representations of language have since been used to model thought disorder and disruptions in semantic connectivity^16,17^. ML-based models have also been developed to classify individuals based on features extracted from verbal fluency (VF) performance, such as word frequency, response time, or semantic similarity^18^. These approaches illustrate how NLP can uncover subtle markers of impaired coherence and concept organization that are difficult to detect through traditional clinical observation. More recently, LLMs have been explored to assist in psychiatric NLP tasks such as annotation and classification of clinical text^19^, while Marinković et al.^20^ applied LLM-based methods to classify verbal fluency responses in Slovene. Together, these studies highlight the expanding role of NLP and AI tools in advancing the objective assessment of linguistic and cognitive disturbances in schizophrenia.

For the Slovene language, Marinković et al.^21^ manually transcribed and analyzed verbal fluency test recordings from healthy controls (HC) and individuals with schizophrenia (SH) to assess speech disorganization, reporting a classification accuracy of 85%. Building on this foundation, the present study introduces a fully automated and explainable processing pipeline that leverages modern ASR systems in combination with LLMs to extract and annotate clinically relevant features directly from raw audio. In this work, LLMs are not used to make clinical decisions directly, but rather to facilitate post-processing and semantic enrichment of linguistic data-enhancing reproducibility, transparency, and reducing manual workload in psychiatric research.

To our knowledge, this study presents the first automated and interpretable system for classifying schizophrenia from Slovene-language verbal fluency (VFT) recordings. Our aim is to develop and evaluate a fully automated, objective pipeline that (i) converts VFT audio to text via ASR, (ii) applies LLM for post-transcription processing, and (iii) trains interpretable ML classifiers. We test the following hypotheses:

- **H1:** The automated pipeline achieves diagnostic performance that is comparable to or better than prior manual approaches on the same dataset, enabling a direct comparison with Marinković et al.^21^.
- **H2:** Automating transcription, annotation, and feature extraction reduces human effort and improves procedural consistency without degrading classification accuracy.
- **H3:** Interpretable models (Naive Bayes, logistic regression, EBM) yield stable, clinically meaningful feature attributions (e.g., totals, error types, timing features) that align with established VFT literature.

**Technical note.** In this paper, we use *artificial intelligence* (AI) as an umbrella term encompassing machine learning (ML) methods for prediction and explanation, and large language models (LLMs) for text processing. ML classifiers (Naive Bayes, logistic regression, EBM) produce the diagnostic predictions from engineered features extracted from audio. LLMs are not used as diagnostic models; rather, they support *post-transcription* processing (minor transcription refinement and semantic annotation of verbal responses). Audio processing covers the pipeline from raw waveforms to automatic speech recognition (ASR), followed by feature extraction tailored to verbal-fluency tasks.

## Methods

### Participants

The study involved 126 Slovene-speaking adults: 68 healthy controls (HC) and 58 individuals with a clinical diagnosis of schizophrenia (SH). Participants in the SH group were recruited and clinically evaluated at the University Psychiatric Clinic Ljubljana, where treating psychiatrists informed eligible patients about the study during regular outpatient visits. Healthy control participants were recruited via announcements at the Faculty of Computer and Information Science, University of Ljubljana. Recruitment aimed to achieve comparable age and gender distribution between groups. All participants were aged 18 years or older and provided written informed consent after receiving a full explanation of the study’s aims and procedures. Participation was voluntary, and all data were anonymized and used solely for research purposes.

To maintain the integrity of the results, specific exclusion criteria were applied. Participants who were unable to speak Slovenian or who had a history of intellectual disability, neurological or organic brain disorders, or substance abuse were excluded. For the healthy control group, additional exclusion criteria included any history of psychiatric disorders or substance use problems. This selection process ensured that observed differences could be attributed to schizophrenia-related cognitive and language alterations rather than other confounding factors.

For all participants, demographic data were collected, including age, gender, education level, school performance, marital status, and employment status. For the schizophrenia group, diagnoses were established by psychiatrists at the University Psychiatric Clinic Ljubljana using the Structured Clinical Interview for DSM-5 (SCID-5) and verified against ICD-10 (In Slovenia, ICD-10 remains the operative diagnostic classification; the nationwide transition to ICD-11 has not yet been implemented. Oversight of this policy rests with the National Institute of Public Health of the Republic of Slovenia (NIJZ).) diagnostic criteria. Clinical metadata were additionally recorded, including illness duration, number of hospitalizations, and co-occurring health conditions. All participants were receiving stable doses of antipsychotic medication at the time of testing. The severity of symptoms varied across participants, ranging from relatively mild manifestations to more pronounced and severe forms of the illness. No participants reported chronic physical illnesses, though 16 individuals mentioned minor unrelated conditions (e.g., seasonal allergies).

Group-level demographic and clinical characteristics are summarized in Table 1. While the participant groups were well-matched in age and gender, statistically significant differences were observed in academic performance, final educational attainment, marital status, and employment status. Given the established effects of schizophrenia on functional outcomes, these disparities likely reflect the consequences of the illness rather than pre-existing group differences.

The data collection process was conducted in compliance with ethical research standards and was approved by the Republic of Slovenia National Medical Ethics Committee under approval number: 0120-51/2024-2711-4. Written informed consent was obtained from all participants before enrollment. The participants were fully briefed on the research objectives and procedures. Data collection and analysis were conducted anonymously to protect participant confidentiality and ensure adherence to ethical guidelines.

**Table 1:** Summary of demographic and clinical characteristics for healthy controls (HC) and participants with schizophrenia (SH).

Measure	HC (N = 68)	SH (N = 58)	Statistical test	p-value
Gender distribution	M: 35, F: 33	M: 29, F: 29	$\chi ^2$	0.869
Median age (years)	46.5	45.0	*t*-test	0.822
Median primary school grade	5.0	3.0	*t*-test	< 0.005
Median high school grade	4.0	3.0	*t*-test	< 0.005
Prevalent education level	Bachelors	High School	$\chi ^2$	< 0.005
Prevalent marital status	Married	Single	$\chi ^2$	< 0.005
Prevalent employment status	Employed	Retired	$\chi ^2$	< 0.005
Median illness duration (years)	–	10.0	–	–
Median hospitalizations	–	4.0	–	–

HC–Healthy controls; SH–Participants with schizophrenia; $\chi ^2$–Chi-squared test; *t*-test–Independent t-test.

### Testing procedure

#### Verbal fluency tasks

Participants completed a verbal fluency assessment consisting of two one-minute subtasks: a phonetic and a semantic task.

In the phonetic task, participants were instructed to produce as many Slovene words as possible beginning with the letter ‘L’, excluding proper nouns such as personal and geographical names.

In the semantic fluency task, participants were asked to name as many animals as possible in Slovene, again excluding pet names and other proper nouns.

Both the phonemic and semantic verbal fluency tasks used in this study are standard components of neuropsychological assessment and are well validated in schizophrenia research. The semantic fluency task is included in widely used cognitive assessment batteries such as the Brief Assessment of Cognition in Schizophrenia (BACS)^22^ and the Repeatable Battery for the Assessment of Neuropsychological Status (RBANS)^23^, which all evaluate verbal production and semantic memory. The phonemic task, requiring participants to generate words beginning with a specific letter, parallels the letter fluency component of the Addenbrooke’s Cognitive Examination (ACE)^24^ and serves as a recognized measure of lexical retrieval and executive control. In our adaptation, the letter ‘L’ was selected for Slovenian due to its high lexical frequency, ensuring comparability with the commonly used F–A–S paradigms in English-language studies. These tasks have demonstrated robust reliability, sensitivity to cognitive disorganization, and strong clinical validity across multiple studies, making them suitable instruments for detecting verbal fluency impairments in schizophrenia.

#### Testing protocol

The testing procedure was standardized for all participants. Each session was conducted individually in a quiet, isolated room to minimize distractions and ensure high-quality audio recordings. Participants sat in front of a computer screen equipped with a microphone and were guided through on-screen instructions. The procedure was self-paced: after reading the instructions, participants initiated each subtask by pressing a key. The phonemic task was always administered before the semantic task, with short breaks allowed between tasks to avoid fatigue. All participants completed the assessment under identical conditions. Healthy controls were tested at the Faculty of Computer and Information Science, University of Ljubljana, while participants with schizophrenia were assessed at the University Psychiatric Clinic Ljubljana.

### Dataset

The final dataset comprises 126 mono-channel audio recordings (one per participant) in WAV format, captured using built-in laptop microphones at a sampling rate of 44.1 kHz with uncompressed PCM encoding. Each recording includes both verbal fluency subtasks. Accompanying CSV files contain timestamp metadata indicating the start and end times of each subtask. These paired audio and timestamp files form the basis for all subsequent transcription and feature extraction steps.

### Audio preprocessing

We implemented audio preprocessing using the pydub Python library^25^.

For each participant, we segmented the original recording into two separate WAV files–corresponding to the phonetic and semantic verbal fluency subtasks-based on timestamp metadata. We then processed these audio segments to enhance consistency and intelligibility across the dataset.

First, we applied downward dynamic range compression using default parameters (threshold = $-20$ dBFS, ratio = 4:1, attack = 5 ms, release = 50 ms) to reduce volume disparities and improve clarity^26^. Next, we normalized the loudness of each segment to a target level of $-20$ dBFS to reduce variability arising from differences in speaker vocal intensity or recording conditions.

### Manual transcription

We obtained manual transcriptions from Marinković et al.^21^, who previously collected and annotated the same verbal fluency recordings. These transcriptions were used solely for evaluating the transcription quality of the ASR models. They were not used in any other part of the processing pipeline, including feature extraction or model training.

### Automated transcription

We applied three ASR systems to generate word-level transcriptions with timestamps for each participant’s audio recording. These transcriptions served as input for all subsequent feature extraction and classification tasks. The evaluated systems were:

- Truebar 24.05^27^, a Slovene-language model accessed via API,
- Whisper large-v3^28^, a multilingual open-source model accessed through the transformers library^29^, and
- Soniox^30^, a commercial multilingual model accessed via WebSocket API.

We used each system to automatically transcribe both the phonetic and semantic audio segments, generating word-level start and end timestamps. To evaluate performance, we compared the outputs against manually annotated reference transcripts using word accuracy (WAcc), defined as $1 - \text {WER}$, where WER denotes the word error rate. We selected the best-performing model based on this comparative evaluation for downstream analysis.

### Post-transcription processing

**Fig. 1:**
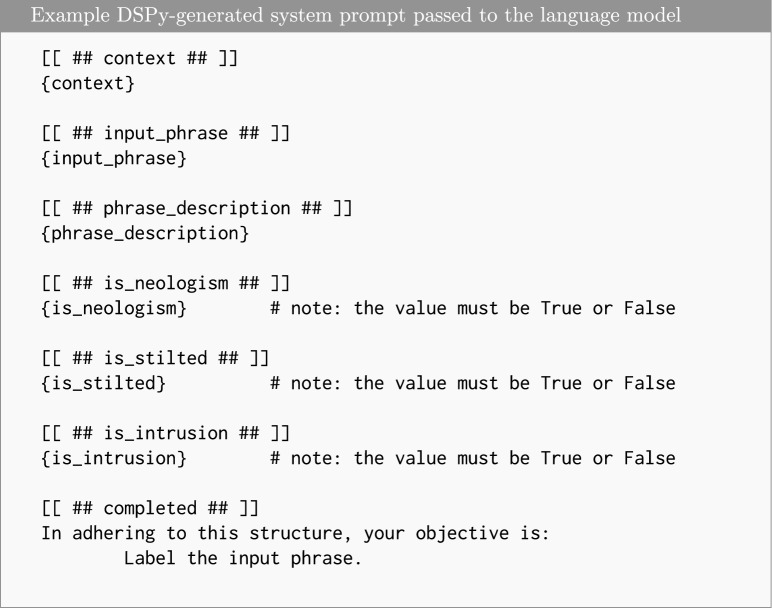
Structured prompt for semantic annotation. Using the provided task context, input phrase, and a phrase description (generated in a preceding step), the model determines whether the word is a neologism, stilted, or an intrusion with respect to the verbal fluency task.

After generating automatic transcriptions, we performed two postprocessing steps: transcription refinement and semantic annotation. Both steps were executed in a fully automated manner using the GPT-4o language model via the DSPy declarative prompting framework^31^, which enables modular, structured, and reproducible prompt design for large-scale text transformation and annotation. An example of the DSPy-generated prompt used is shown in Fig. 1.

Transcription refinement was performed using a two-stage pipeline to improve the quality and task relevance of the ASR-generated transcriptions. In the first stage, the model filtered out words that violated verbal fluency task constraints–such as filler words, personal comments, or off-topic content–retaining only responses consistent with the task instructions. In the second stage, the model contextually corrected clearly misrecognized words while preserving ambiguous or potentially novel expressions (e.g., neologisms or uncommon lexical items). The entire process was fully automated and involved no manual intervention.

Semantic annotation was then applied to the refined transcripts using additional LLM-based processing. For each word, the model automatically assessed several task-specific semantic and pragmatic features, including whether the word constituted an intrusion (i.e., not semantically appropriate for the category), whether it exhibited stilted or overly formal usage, and whether it was a neologism or otherwise absent from standard Slovene lexicons. For responses in the semantic fluency task (i.e., animal naming), the model additionally generated a page-long descriptive paragraph summarizing the animal’s appearance, habitat, and behavior. For intrusions and neologisms, it produced similarly detailed contextual explanations, outlining possible meanings, referents, or usage scenarios.

### Feature engineering

Based on the refined and semantically annotated transcriptions, we engineered a total of 56 features, grouped into two categories: verbal and non-verbal.

We derived verbal features directly from the transcriptions generated by automatic speech recognition (ASR) models and organized them into three groups: temporal, semantic, and phonetic features.

Temporal features captured speech dynamics such as production speed and inter-word pauses. Semantic features quantified anomalies and task relevance, including the proportion of neologisms, stilted expressions, and intrusions. Phonetic features reflected the extent of post-processing corrections, based on the assumption that disorganized or impoverished speech typically reduces ASR quality.

We extracted non-verbal features from speech spectrograms using two toolkits: OpenSMILE^32^ and DisVoice^33^. We retained only those features robust to room conditions, focusing on vocal descriptors such as pitch, jitter, shimmer, and pitch perturbation quotient.

The final feature configuration included 39 verbal features (20 temporal, 17 semantic, and 2 phonetic), 17 non-verbal features (14 OpenSMILE and 3 DisVoice), and 2 demographic variables (age and gender), resulting in a total of 58 features. For a comprehensive overview of all extracted features, refer to Supplementary Table S1 (verbal features) and Supplementary Table S2 (non-verbal features).

### Machine learning classification

We trained and evaluated six classification algorithms: Naive Bayes, regularized logistic regression with L1 (Lasso) and L2 (Ridge) penalties, Explainable Boosting Machine (EBM), Random Forest, Support Vector Classifier (SVC) with a radial basis function kernel, and XGBoost.

The first three models–Naive Bayes, logistic regression, and EBM–were treated as interpretable (‘white-box’) approaches and prioritized for explanation and interpretation of results. The remaining models were included to assess whether restricting the analysis to interpretable methods would incur a significant performance trade-off.

Each model was trained separately on three feature sets: verbal (V), non-verbal (N), and combined (VN). All features were Z-score normalized prior to training. All models were trained using their default hyperparameters without further optimization. This decision was made to avoid partitioning our limited dataset, allowing us to use all available data for model training.

Model performance was evaluated solely within the current dataset using leave-one-out cross-validation (LOOCV). The evaluation metrics included classification accuracy (CA), sensitivity, specificity, positive predictive value (PPV), F1 score, and area under the ROC curve (AUC), using a fixed decision threshold of 0.5.

Based on AUC, we selected the best-performing interpretable model for further analysis using visual global and local explanation methods appropriate to its architecture. To isolate the effect of potential automated transcription errors, the selected model was additionally retrained and evaluated on manually transcribed dataset. This allowed for a direct comparison of model performance on lexically perfect data versus the data derived from the automated transcription workflow.

## Results

### Qualitative analysis of ASR transcriptions

The comparison of transcription accuracy between manual annotations and three ASR models–Truebar, Soniox, and Whisper–is presented in Table 2.

At the transcription level, we observe a substantial improvement in WAcc from the unprocessed (U) to the filtered (F) transcriptions, with gains ranging from 0.18 to 0.39. The subsequent refinement from filtered to adjusted (A) yields smaller gains between 0.01 and 0.09. This pattern suggests that the largest improvements are achieved by filtering irrelevant or malformed content, while further adjustments provide only marginal benefit–particularly for higher-quality models.

At the group level, all ASR models consistently performed better on speech from healthy controls than on speech from individuals with schizophrenia. Across all systems and transcription levels, the probability that the mean WAcc is higher for the HC group was $\hat{p}_\mu = 1.00$, indicating that, within the limits of posterior sample resolution, we are as statistically certain as possible that ASR models, on average, perform better on healthy speech than on speech impaired by schizophrenia.

At the model level, Truebar demonstrated the highest overall transcription quality. Specifically, it achieved the best WAcc for both healthy individuals ($\bar{\mu }_h = 0.75$) and participants with schizophrenia ($\bar{\mu }_s = 0.52$) at the adjusted level. These results led to the selection of Truebar as the default model for downstream processing.

Although Whisper showed the strongest discriminative potential between groups ($\hat{p}_r = 0.78 \pm 0.01$), its overall transcription accuracy was considerably lower. Consequently, its outputs were deemed unsuitable for reliable downstream feature extraction and classification.

**Table 2:** Comparison of word accuracy (WAcc) between manual and ASR transcriptions for healthy controls (HC) and individuals with schizophrenia (SH), across transcription levels: unprocessed (U), filtered (F), and adjusted (A), grouped by ASR model.

ASR model: Truebar						
Metric	HC		SH		HC vs. SH	
	$\bar{\mu _h}$	95% HDI	$\bar{\mu _s}$	95% HDI	$\hat{p_\mu }$	$\hat{p_r} \pm SE$
WAcc (U)	0.49	[0.31, 0.45]	0.24	[0.17, 0.31]	$\boldsymbol{1.00}$	$0.64 \pm 0.01$
WAcc (F)	0.73	[0.68, 0.78]	0.49	[0.41, 0.56]	$\boldsymbol{1.00}$	$0.74 \pm 0.01$
WAcc (A)	$\boldsymbol{0.75}$	[**0.70, 0.79**]	$\boldsymbol{0.52}$	[**0.45, 0.59**]	$\boldsymbol{1.00}$	$0.73 \pm 0.01$

ASR model: Soniox						
Metric	HC		SH		HC vs. SH	
	$\bar{\mu _h}$	95% HDI	$\bar{\mu _s}$	95% HDI	$\hat{p_\mu }$	$\hat{p_r} \pm SE$
WAcc (U)	0.27	[0.20, 0.33]	0.13	[0.08, 0.18]	$\boldsymbol{1.00}$	$0.65 \pm 0.01$
WAcc (F)	0.66	[0.61, 0.71]	0.43	[0.37, 0.51]	$\boldsymbol{1.00}$	$0.72 \pm 0.01$
WAcc (A)	0.67	[0.62, 0.73]	0.45	[0.37, 0.52]	$\boldsymbol{1.00}$	$0.72 \pm 0.01$

ASR model: Whisper						
Metric	HC		SH		HC vs. SH	
	$\bar{\mu _h}$	95% HDI	$\bar{\mu _s}$	95% HDI	$\hat{p_\mu }$	$\hat{p_r} \pm SE$
WAcc (U)	0.20	[0.14, 0.25]	0.07	[0.04, 0.10]	$\boldsymbol{1.00}$	$0.66 \pm 0.01$
WAcc (F)	0.51	[0.45, 0.56]	0.25	[0.19, 0.31]	$\boldsymbol{1.00}$	$\boldsymbol{0.78 \pm 0.01}$
WAcc (A)	0.60	[0.54, 0.65]	0.32	[0.25, 0.39]	$\boldsymbol{1.00}$	$0.77 \pm 0.01$

For each metric, the highest result is highlighted in bold; in case of ties, multiple values are bolded.

WAcc–Word accuracy; ASR–Automatic speech recognition; HC–Healthy controls; SH–Participants with schizophrenia; HDI–Highest density interval; SE–Standard error. Probability metrics:  $\hat{p}_\mu$–Probability that the group-level posterior mean is higher for HC than SH;  $\hat{p}_r$–Probability that a randomly selected HC outperforms a randomly selected SH based on posterior predictive samples. Transcription levels: U–Unprocessed; F–Filtered; A - Adjusted.

### Comparison of manual and automated post-transcription processing

To evaluate our automated post-transcription processing, we compared its output against a manually processed data for both transcription refinement and semantic tagging. The results of this comparison are summarized in Table 3.

In standard VF administration, manual transcripts contain only task-eligible responses. Examiners do not document fillers (e.g., “uh”, “um”), comments, questions to the examiner, or other non-task speech, in accordance with neuropsychological scoring guidelines^34,35^. The manual transcripts in our dataset are response-only records, making a direct comparison of filtering and correction rates unfeasible. We report these metrics only for the automated processing.

The data shows that the automated processing filtered a higher proportion of words from HC during the phonetic task, while more words were filtered from the SH group during the semantic task. This divergence likely reflects the tendency of HCs to produce filler words when exhausting the constrained phonetic task, while their robust lexical access allows for more fluent production during the less constrained semantic task. Notably, the overlap between words that were filtered and those that required correction was substantially higher for the HC group in both tasks ($\approx$ 95% vs. $\approx$ 80%). This suggests that the speech from healthy individuals contained fewer distinct types of errors, which is consistent with clearer and more task-focused speech production.

For semantic tagging, a direct comparison of intrusion and neologism rates was possible. The GPT-4o-based system consistently identified a higher percentage of both intrusions and neologisms than the manual annotator. The highest relative differences were observed for semantic intrusions in HC, where rates increased from 0.09% (manual) to 2.78% (automated), corresponding to an average increase from approximately 0.02 to 0.6 intrusions per semantic task. Overall, the relative differences between the HC and SH groups were directionally consistent across both methods, indicating that the automated system reliably captures the same underlying error patterns as a human expert.

**Table 3:** Comparison of automated (GPT-4o) and manual annotation for transcription refinement and semantic tagging.

Metric	Phonetic task		Semantic task	
	HC	SH	HC	SH
Transcription refinement (automated only)				
Retention rate: U vs. F (%)	63.06	**82.64**	**84.59**	72.90
Overlap: F vs. A (%)	**94.81**	79.56	**94.55**	80.50
Semantic tagging				
Manual Intrusions (%)	3.06	**11.17**	0.09	**2.05**
Automated Intrusions (%)	9.31	**20.65**	2.78	**8.53**
Manual Neologisms (%)	0.00	**2.54**	0.00	**0.39**
Automated Neologisms (%)	0.16	**0.93**	0.00	**1.27**

Significant values are in [bold].

HC–Healthy controls; SH–Schizophrenia patients.

Transcription levels: U–Unprocessed; F–Filtered; A - Adjusted.

### Feature-based statistical differences

We identified the ten most discriminative features based on discriminative deviation (DD), defined as the absolute deviation from chance ($|\hat{p}_r - 0.5|$), which quantifies each feature’s ability to differentiate between HC and SH. We report these features in Table 4.

Across all top-ranked features, we observed consistent group-level differences. In nearly all cases, the posterior probability that the mean feature value differs between HC and SH was $\hat{p}_\mu = 1.00$ or $\hat{p}_\mu = 0.00$, reflecting the highest possible certainty given the posterior sampling resolution. The only exception was feature V30, which still indicated a very high degree of certainty with $\hat{p}_\mu = 0.99$.

The most discriminative feature at the subject level was feature V1, which measures the rate of phrase production during the semantic fluency task. HC produced more utterances per second than SH, with average rates of $\bar{\mu }_h = 0.36$ and $\bar{\mu }_s = 0.19$, respectively. Subject-level posterior comparisons indicated that in 83% of random pairings, an HC participant exhibited a higher phrase rate than an SH participant ($\hat{p}_r = 0.83 \pm 0.01$).

The second most discriminative feature (V3), quantifies the longest pause during the semantic task. The mean maximum gap occupied 44% of the task duration in SH ($\bar{\mu }_s = 0.44$), compared to 20% in HC ($\bar{\mu }_h = 0.20 \pm 0.01$). Related temporal features V5 and V6 showed similar discriminative relevance, ranking 5th and 6th respectively, further highlighting the importance of temporal speech characteristics in distinguishing between HC and SH.

Several top-ranked non-verbal features (N3, N8, and N10) captured prosodic properties of speech, specifically characterizing the mean and variability of F0 (fundamental frequency) semitone slopes, which reflect pitch dynamics. Compared to SH, HC exhibited higher rising slopes ($\bar{\mu }_h = 405.84 \pm 0.51$ vs. $\bar{\mu }_s = 212.20 \pm 0.43$) and greater variability in both rising ($\bar{\mu }_h = 277.60 \pm 0.30$ vs. $\bar{\mu }_s = 169.71 \pm 0.23$) and falling ($\bar{\mu }_h = 281.42 \pm 0.36$ vs. $\bar{\mu }_s = 177.05 \pm 0.43$) pitch contours. These differences likely reflect reduced pitch modulation and prosodic flattening in individuals with schizophrenia.

Features V27 and V30 reflect speech coherence during the phonetic task. These features capture the average and minimum phonetic similarity between consecutive utterances, approximated using Levenshtein distance. Healthy controls produced more phonologically similar sequences on average ($\bar{\mu }_h = 0.29$, $\bar{\mu }_s = 0.19$) and exhibited fewer incoherent transitions ($\bar{\mu }_h = 0.14$, $\bar{\mu }_s = 0.09$), whereas responses from individuals with schizophrenia were more phonologically dispersed.

Lastly, feature V39 quantifies the Levenshtein similarity between filtered and adjusted transcriptions. Healthy controls showed higher similarity scores ($\bar{\mu }_h = 0.97$) compared to individuals with schizophrenia ($\bar{\mu }_s = 0.89$), suggesting that transcripts from the SH group required more post-processing corrections. This likely reflects greater irregularities or distortions in speech production, which reduce ASR reliability and increase the need for additional adjustments.

**Table 4:** Group-level comparison of the top features between healthy controls (HC) and individuals with schizophrenia (SH), ordered by descending discriminative deviation (DD).

Feature ID	HC		SH		HC vs. SH		
	$\bar{\mu _h}$	95% HDI	$\bar{\mu _s}$	95% HDI	$\hat{p_\mu }$	$\hat{p_r} \pm SE$	DD
V1	0.36	[0.33, 0.39]	0.19	[0.15, 0.23]	1.00	$0.83 \pm 0.01$	0.33
V3	0.20	[0.17, 0.23]	0.44	[0.38, 0.50]	0.00	$0.23 \pm 0.01$	0.27
V16	0.21	[0.18, 0.23]	0.10	[0.08, 0.12]	1.00	$0.76 \pm 0.01$	0.26
N10	405.84	[345.60, 468.71]	212.20	[160.06, 262.52]	1.00	$0.75 \pm 0.01$	0.25
V5	0.05	[0.04, 0.06]	0.12	[0.10, 0.15]	0.00	$0.26 \pm 0.01$	0.24
V6	0.04	[0.04, 0.04]	0.24	[0.18, 0.30]	0.00	$0.26 \pm 0.01$	0.24
V27	0.29	[0.27, 0.32]	0.19	[0.14, 0.24]	1.00	$0.73 \pm 0.01$	0.23
N3	277.60	[243.12, 314.33]	169.71	[141.11, 197.86]	1.00	$0.73 \pm 0.01$	0.23
V30	0.14	[0.12, 0.16]	0.09	[0.06, 0.12]	0.99	$0.73 \pm 0.01$	0.23
V39	0.97	[0.96, 0.98]	0.89	[0.86, 0.92]	1.00	$0.72 \pm 0.01$	0.22
N8	281.42	[239.80, 324.92]	177.05	[129.23, 228.00]	1.00	$0.71 \pm 0.01$	0.21

HC–healthy controls; SH–individuals with schizophrenia; HDI–highest density interval; SE–standard error; DD–discriminative deviation. Probability metrics:  $\hat{p}_\mu$–Probability that the group-level posterior mean is higher for HC than SH;  $\hat{p}_r$–Probability that a randomly selected HC outperforms a randomly selected SH based on posterior predictive samples. Feature types: V{n}–Verbal features; N{n}–Non-verbal features.

### Machine learning results

We summarize the performance of all models across verbal (V), non-verbal (N), and combined (VN) feature configurations in Table 5. On average, models trained separately on verbal and non-verbal feature sets achieved comparable performance, with both yielding an average AUC of 0.83. When we combined verbal and non-verbal features, performance improved consistently across all metrics, increasing the average AUC to 0.86. Additional metrics for the combined feature set include a classification accuracy (CA) of 0.76, sensitivity (SEN) of 0.69, specificity (SPE) of 0.82, positive predictive value (PPV) of 0.76, and F1 score of 0.73.

The highest overall performance was achieved by the Explainable Boosting Machine (EBM) trained on the combined feature set. The EBM reached a classification accuracy of 0.82, sensitivity of 0.76, specificity of 0.87, PPV of 0.83, F1 score of 0.79, and an AUC of 0.90.

Given its strong performance and inherent interpretability, we selected the EBM trained on the combined feature set for subsequent global and local explanation analyses.

**Table 5:** LOOCV performance of the evaluated models in predicting schizophrenia across different feature configurations.

Model	Feature set	CA	SEN	SPE	PPV	F1	AUC
GNB	V	0.74	0.55	**0.90**	0.82	0.66	0.78
	N	0.71	0.53	0.85	0.76	0.63	0.81
	VN	0.74	0.55	**0.90**	0.82	0.66	0.81
Lasso	V	0.79	0.74	0.84	0.80	0.77	0.85
	N	0.73	0.67	0.78	0.72	0.70	0.85
	VN	0.72	0.69	0.75	0.70	0.70	0.86
Ridge	V	0.74	0.67	0.79	0.74	0.70	0.77
	N	0.71	0.66	0.75	0.69	0.67	0.78
	VN	0.71	0.62	0.79	0.72	0.67	0.79
EBM	V	0.78	0.71	0.84	0.79	0.75	0.86
	N	0.77	0.72	0.81	0.76	0.74	0.84
	VN	**0.82**	**0.76**	0.87	**0.83**	**0.79**	**0.90**
RF	V	0.74	0.67	0.79	0.74	0.70	0.84
	N	0.78	0.72	0.82	0.78	0.75	0.84
	VN	0.79	0.72	0.85	0.81	0.76	0.89
SVC	V	0.78	0.72	0.82	0.78	0.75	0.83
	N	0.71	0.66	0.76	0.70	0.68	0.82
	VN	**0.82**	**0.76**	0.87	**0.83**	**0.79**	0.87
XGBoost	V	0.72	0.69	0.75	0.70	0.70	0.82
	N	0.75	0.76	0.75	0.72	0.74	0.85
	VN	0.73	0.72	0.74	0.70	0.71	0.88
Average	V	0.75	0.68	0.81	0.77	0.72	0.83
	N	0.74	0.67	0.79	0.74	0.70	0.83
	VN	0.76	0.69	0.82	0.76	0.73	0.86

For each metric, the highest result is highlighted in bold; in case of ties, multiple values are bolded.

CA–Classification accuracy; SEN–sensitivity; SPE–specificity; PPV–Positive Predictive Value; F1–F1 score; AUC–Area Under the ROC Curve. Model abbreviations. GNB–Gaussian Naive Bayes; Lasso–L1-regularized Logistic Regression; Ridge–L2-regularized Logistic Regression; EBM–Explainable Boosting Machine; RF–Random Forest; SVC–Support Vector Classifier; XGBoost–Extreme Gradient Boosting. Feature sets. V–Verbal; N–Non-verbal; VN–Verbal + Non-verbal combined.

### Interpretation of EBM

EBM extends generalized additive models (GAMs) by learning feature-wise contributions in an additive structure. Its architecture is inherently transparent by design, learning a separate function (shape function) for each feature and then summing their individual contributions to form a final prediction.

Each shape function is learned independently using gradient boosting algorithm. The weak learners in this process are shallow decision trees, but with the critical constraint that each tree is trained using only a single feature at a time. While an individual tree provides a simple, discrete step, the boosting process combines thousands of these trees. This summation results in a smooth, continuous, and potentially highly non-linear shape function for each feature, allowing the model to capture complex relationships in the data without sacrificing interpretability.

For this binary classification task, the model is specified as:

1 $$\begin{aligned} \text {logit}(\mathbb {P}[y = 1]) = \beta _0 + \sum _i f_i(x_i), \end{aligned}$$

where $\beta _0$ is the learned intercept, and $f_i(x_i)$ is the shape function representing the contribution of feature *i*. No interaction terms were included to preserve interpretability. Final probabilities are obtained by applying the inverse-logit (sigmoid) function.

#### Global interpretation

The primary advantage of this additive, single-feature approach is its direct interpretability. Because each shape function is learned independently, the global effect of any feature can be understood by visualizing a plot of its shape function against its corresponding feature values. To quantify this, the global importance of a feature is calculated as the average absolute contribution across all samples:

2 $$\begin{aligned} \text {Importance}_i = \frac{1}{n} \sum _{k=1}^{n} \left| f_i(x_{k,i}) \right| , \end{aligned}$$

where *n* is the number of samples, $x_{k,i}$ is the value of feature *i* for sample *k*, and $f_i(x_{k,i})$ is its corresponding contribution.

**Fig. 2:**
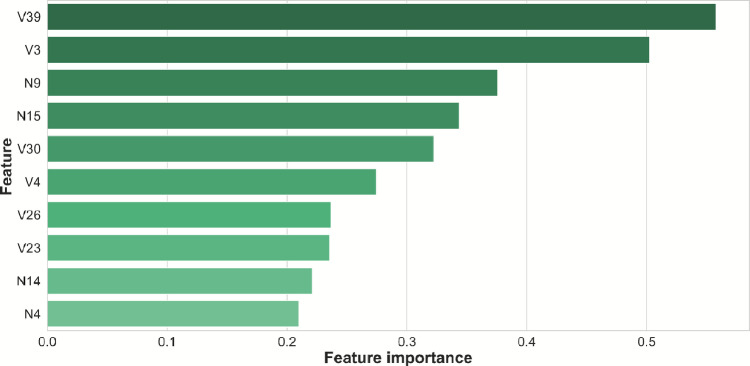
Global feature importance plot showing the top 10 most influential features for the EBM model trained on the combined feature set. Features are displayed by their IDs; for a detailed description of each feature, see Supplementary Table S1 (verbal features) and Supplementary Table S2 (non-verbal features).

The ten most important features, based on average absolute contribution, are shown in Fig. 2. Three of these features (V3, V30, and V39) overlap with the top-ranked features identified in section “Feature-based statistical differences”, reinforcing their robustness across both statistical and model-based analyses.

Feature V4 is a temporal measure that represents the minimum gap between consecutive utterances, further highlighting the importance of pacing and pause-related dynamics in distinguishing speech patterns between groups. Features V23 and V26 capture, respectively, the maximum semantic similarity between consecutive words and the kurtosis of semantic similarity scores during the semantic task–underscoring both lexical fluency and consistency as key indicators of organized speech.

The remaining features are non-verbal. Two are prosodic F0-based measures: the standard deviation of normalized F0 across both tasks (N4) and the F0 0–2 percentile range (N9), both reflecting low-frequency pitch variation. The other two are voice quality features: the mean phonation perturbation quotient (N15) and the standard deviation of shimmer in decibels (N14), also averaged across tasks. These findings suggest that while prosodic and voice quality features are less discriminative in isolation, they contribute meaningfully in multivariate predictive models by capturing complementary acoustic signatures of disorganized or effortful speech.

#### Local interpretation

**Fig. 3:**
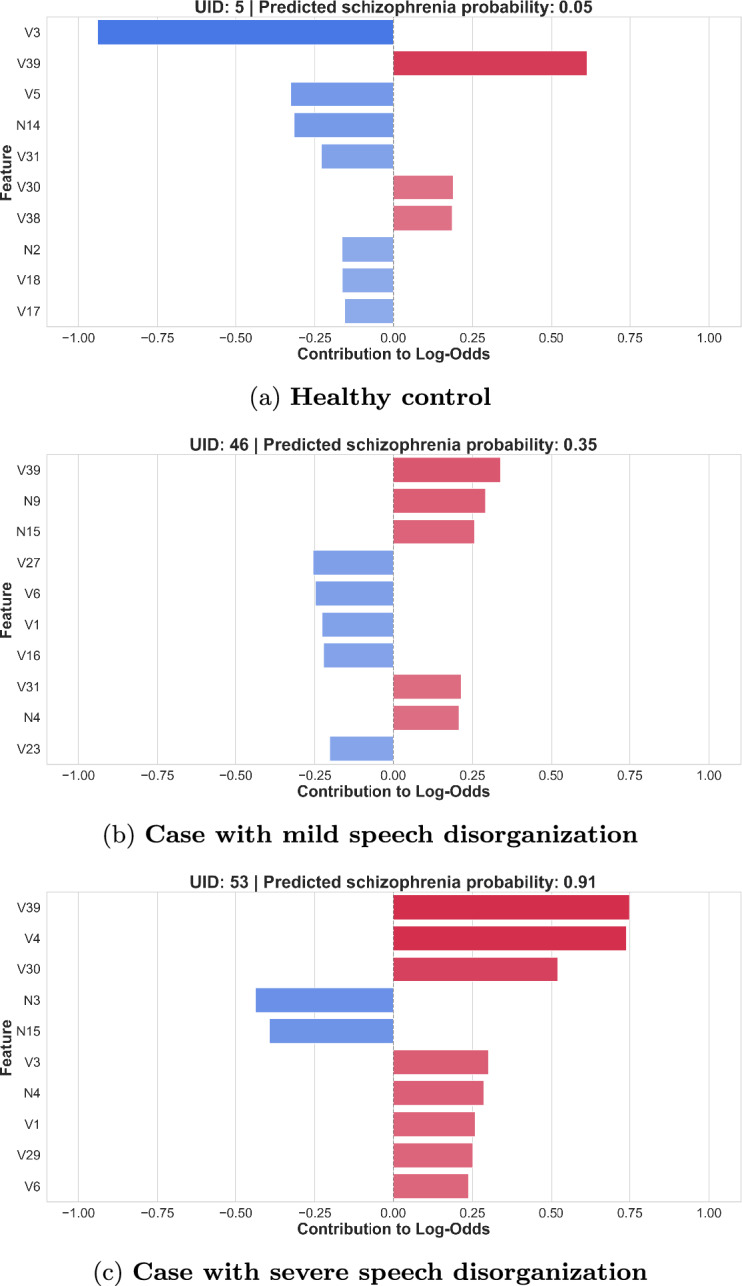
Local feature importance plots for three representative individuals–a healthy control (**a**), a participant with speech patterns indicative of mild disorganization (**b**), and a participant with patterns indicative of severe disorganization (**c**)–as predicted by the EBM model. The plots are based on the 10 most important features for each respective case. Red bars indicate contributions increasing the log-odds toward the schizophrenia class, while blue bars indicate contributions decreasing the log-odds toward the healthy control class. For a detailed description of each feature, see Supplementary Table S1 (verbal features) and Supplementary Table S2 (non-verbal features).

Local interpretation is derived from the additive decomposition defined in Eq. (1), where each feature’s contribution $f_i(x_i)$ represents its signed effect on the log-odds of classification. Positive contributions increase the likelihood of a schizophrenia classification, while negative values shift the prediction toward the healthy control class. Figure 3 illustrates local feature contributions for three representative individuals–a healthy control, a participant with speech patterns indicative of mild disorganization, and a participant with patterns indicative of severe disorganization–demonstrating how the model integrates feature-level evidence to generate individualized predictions.

Local interpretability analysis showed that the order and contribution of the most influential features varied across individuals, indicating that predictions are driven by subject-specific feature patterns.

For the healthy control, the predicted probability of schizophrenia was 0.04. Based on the visual representation, feature V3 had the strongest overall impact, pushing the prediction toward the healthy class. The second most influential feature, V39, pointed toward the schizophrenia class. The remaining features had substantially smaller individual effects, however, the vast majority supported the healthy class. As a result, despite one strong opposing feature, the overall probability of schizophrenia remained very low.

For the participant with speech patterns indicative of mild disorganization, feature contributions were mixed, with approximately equal influence from features supporting each class. The resulting predicted probability was 0.30, representing a false negative under the default 0.5 threshold.

For the participant with speech patterns indicative of severe disorganization, nearly all top-ranked features contributed toward the schizophrenia class, producing a predicted probability of 0.91.

These cases illustrate how the model incorporates individual-specific patterns in prediction and how local explanations can clarify which features influenced the final classification.

### Comparison of EBM performance on automated vs. manual transcripts

To isolate the effect of potential transcription errors, we benchmarked our best-performing EBM from the automated workflow against a version of the model retrained on the manually transcribed data. Retraining on the manual data necessarily excluded the phonetic features, as these are artifacts of the automated workflow that quantify post-processing—a step not applicable to manually curated transcripts. The model trained on the automated transcripts achieved slightly higher performance across all metrics compared to the model trained on the manual data (Table 6).

**Table 6:** Performance of models trained on automated vs. manual transcripts.

	CA	SEN	SPE	PPV	F1	AUC
Automated	**0.82**	**0.76**	**0.87**	**0.83**	**0.79**	**0.90**
Manual	0.80	0.74	0.85	0.81	0.77	0.85

Significant values are in [bold].

CA–classification accuracy; SEN–sensitivity; SPE–specificity; PPV–Positive Predictive Value; F1–F1 score; AUC–Area Under the ROC Curve.

A likely explanation for this performance decrease on the lexically perfect data is the exclusion of these highly predictive phonetic features, one of which was the Levenshtein distance between the filtered and adjusted transcriptions. Our global interpretation identified this feature as the single most important predictor in the original model. This finding suggests that the information lost by omitting these process-derived features may be greater than the information gained from achieving perfect lexical accuracy.

## Discussion

This study presents a fully automated and transparent machine learning framework for detecting schizophrenia from verbal fluency test recordings conducted in the Slovene language. The pipeline integrates audio preprocessing, automatic speech transcription and annotation, and the extraction of verbal and non-verbal features aligned with clinically relevant symptom domains. We trained classification models using a leave-one-out cross-validation protocol. The best-performing model (EBM), achieved strong predictive performance (AUC = 0.90, CA = 0.82) on a balanced dataset of 126 participants.

Our results demonstrate that combining verbal and non-verbal features consistently improves classification performance compared to using either modality alone. Importantly, the EBM model enables transparent interpretation of both global feature importance and individual-level predictions–an essential property in clinical settings, where explainability supports trust, accountability, and integration into decision-making workflows. Furthermore, the features identified as most important by the EBM-such as temporal dynamics (e.g., pause duration; V4), semantic coherence (e.g., similarity between words; V23, V26), prosodic variation (e.g., pitch range; N4, N9), and voice quality (e.g., shimmer and perturbation; N14, N15)-are consistent with established VFT literature on schizophrenia, supporting our third hypothesis (H3).

Compared to previous automated approaches for speech-based schizophrenia detection, our method achieves competitive classification performance while offering substantially greater explainability. Prior studies have demonstrated the feasibility of automated classification, while generally prioritizing performance over interpretability. For instance, studies in English by Xu et al. reported classification accuracies from 69–75% using a combination of verbal and non-verbal features, later improving to 82% with the addition of semantic coherence measures^10,11^. In Dutch, Ciampelli et al.^12^ reached 77% accuracy using an ASR and semantic NLP approach. Other work, also in English, by Chakraborty et al.^9^ reported accuracies of 79–86% using low-level acoustic features to predict negative symptoms. Although effective, these methods either use opaque feature representations or inherently unexplainable model architectures. In contrast, our approach uses clinically grounded, handcrafted verbal features aligned with DSM-5 and ICD-10 criteria, and interpretable non-verbal features such as jitter, shimmer, and pitch perturbation quotient. Explainable Boosting Machine (EBM) is a transparent machine learning model that enables clear inspection of feature importance and decision logic. Despite prioritizing explainability, our model achieves performance comparable to state-of-the-art systems in other languages, and closely matches the results reported by Marinković et al.^21^ (CA = 0.85, AUC = 0.86), who used the same dataset but relied on manual transcription and feature annotation. A finding that directly supports our first hypothesis (H1).

While the raw transcription accuracy of the selected ASR system was modest-75% for healthy controls and 52% for individuals with schizophrenia-our findings demonstrate that this is not a limitation but rather a source of powerful predictive information. This conclusion is empirically supported by our benchmark analysis, where the EBM model trained with features from the automated workflow slightly outperformed the same model trained on manually transcribed data (AUC 0.90 vs. 0.85).

The explanation we provide in section “Comparison of EBM performance on automated vs.manual transcripts” implies that systematic ASR errors—driven by disfluencies, atypical prosody, or articulation difficulties characteristic of schizophrenia—carry additional diagnostic information that would not be present in perfectly accurate lexical transcriptions. In practice, some of our predictive features therefore depend not only on the underlying speech production, but also on how the ASR system responds to that speech: the “noise” in the transcript, and our post-processing (phonetic) features that explicitly quantify it, both reflect the degree to which the signal deviates from typical fluent speech. This dual dependence can reduce transparency regarding the precise clinical mechanisms encoded by a given feature, even though the ASR-induced noise itself is informative about symptomatology. Although our feature set was carefully designed to capture symptom dimensions grounded in established diagnostic frameworks (DSM-5, ICD-10), interpretations must be made with the awareness that part of the predictive signal arises from properties of the automated transcription process as well as from the acoustic–linguistic characteristics of the speech itself.

Several other limitations should also be acknowledged. While our sample size is adequate for exploratory modeling, further validation on larger and more demographically diverse clinical populations is needed to confirm generalizability. In addition, future work should evaluate the model’s ability to distinguish schizophrenia from clinically related conditions–such as depression or bipolar disorder–which may exhibit overlapping speech characteristics. Variability in recording environments between control and patient groups may also introduce confounding acoustic artifacts. These effects should be minimized in future studies through standardized recording protocols and more controlled data collection procedures, enabling more rigorous testing of the model’s diagnostic specificity across a broader spectrum of psychiatric disorders.

Our proposed framework is automated, cost-effective, and suitable for real-time implementation. It achieves classification performance comparable to state-of-the-art automated systems in other languages and closely replicates the results obtained through manual transcription and feature annotation on the same dataset, while remaining fully transparent. By achieving this performance without manual intervention, our work confirms our second hypothesis (H2). We believe that this combination of accuracy, scalability, and explainability makes the methodology a promising candidate for future integration into clinical workflows and provides a solid foundation for ongoing research in computational psychiatry.

From a clinical standpoint, our approach shows strong potential as a complementary decision-support tool for clinical psychiatrists. It provides objective assessment of speech disorganization, which is a key symptom dimension in schizophrenia. Since verbal-fluency tasks are already a part of routine neuropsychological assessment, the model could be integrated into existing workflows for screening, longitudinal monitoring, and evaluation of treatment response. The simplicity of the task and the fully automated pipeline further support use in low-cost and/or remote settings.

Although the present study was conducted with Slovene-speaking participants, the methodology is in principle language-agnostic. Provided that reliable automatic speech recognition (ASR) and large language model (LLM) resources exist for a target language, comparable performance should be expected. Formal cross-linguistic validation will be essential to confirm robustness across diverse populations and recording conditions.

## Supplementary Information

Supplementary Information.

## Data Availability

The datasets generated and/or analyzed during the current study are not publicly available due to restrictions by Republic of Slovenia National Medical Ethics Committee (approval number 0120-51/2024-2711-4) but are available from the cor- responding author on reasonable request and in agreement with the above Medical Ethics Committee.

## Code availability

The underlying code for this study, along with ASR transcriptions and the training and validation sets, is available in rokrajher/automated-schizophrenia-detection and can be accessed via this link: https://github.com/rokrajher/automated-schizophrenia-detection.
