# Supplementary material for: Automated speech-fluency explanations for schizophrenia diagnosis
Source: Sci Rep. 2025 Dec 22;16:3243. doi: 10.1038/s41598-025-33129-w (PMC12830904; doi:10.1038/s41598-025-33129-w)
Supplement: Supplementary file 1 — Supplementary Information. [file 41598_2025_33129_MOESM1_ESM.pdf]

Supplementary material

Table S1: List of verbal (V) features with their descriptions.

Feature ID	Description
V1	Number of phrases produced per second (semantic task)
V2	Index of pressured speech, reflecting the frequency of rapid consecutive utterances (semantic task)
V3	Maximum silent gap between utterances, expressed as a percentage of the total task duration (semantic task)
V4	Minimum silent gap between utterances, expressed as a percentage of the total task duration (semantic task)
V5	Standard deviation of silent gaps between utterances, expressed as a percentage of task duration (semantic task)
V6	Mean silent gap between utterances, expressed as a percentage of task duration (semantic task)
V7	Speech rate measured in letters per second (semantic task)
V8	Skewness of the distribution of silent gap durations, expressed on percentage scale (semantic task)
V9	Kurtosis of the distribution of silent gap durations, expressed on percentage scale (semantic task)
V10	Linear trend (slope) of gap durations across the task timeline (semantic task) obtained by least squares error fit
V11	Gap trend difference between the tasks (semantic - phonetic)
V12	Kurtosis gap difference between the tasks (semantic - phonetic)
V13	Maximum gap percent difference between the tasks (semantic - phonetic)
V14	Mean gap percent difference between the tasks (semantic - phonetic)
V15	Minimum gap percent difference between the tasks (semantic - phonetic)
V16	Phrases-per-second difference between the tasks (semantic - phonetic)
V17	Pressured speech index difference between the tasks (semantic - phonetic)
V18	Skewness gap difference between the tasks (semantic - phonetic)
V19	Speech speed difference between the tasks (semantic - phonetic)
V20	Standard deviation gap percent difference between the tasks (semantic - phonetic)

Continued on next page

Table S1 – continued from previous page

Feature ID	Description
V21	Average semantic coherence index, computed as the mean cosine similarity between full-page descriptions of consecutive utterances (semantic task)
V22	Standard deviation of semantic coherence (semantic task)
V23	Maximum semantic coherence (semantic task)
V24	Minimum semantic coherence (semantic task)
V25	Skewness of semantic coherence (semantic task)
V26	Kurtosis of semantic coherence (semantic task)
V27	Average phonetic coherence, computed as the mean Levenshtein similarity between consecutive utterences (phonetic task)
V28	Standard deviation of phonetic coherence (phonetic task)
V29	Maximum phonetic coherence (phonetic task)
V30	Minimum phonetic coherence (phonetic task)
V31	Skewness of phonetic coherence (phonetic task)
V32	Kurtosis of phonetic coherence (phonetic task)
V33	Information index, calculated as $1 - V34 - V35$, averaged across both VF tasks
V34	Percentage of intrusions averaged across both VF tasks
V35	Percentage of neologisms averaged across both VF tasks
V36	Percentage of stilted or overly formal expressions averaged across both VF tasks
V37	Repetition index, calculated as a normalized weighted measure of word repetitions, averaged across both VF tasks.
V38	Word accuracy between unprocessed and filtered transcription levels, averaged across both VF tasks
V39	Levenshtein similarity between filtered and adjusted transcription levels, averaged across both VF tasks

Table S2: List of non-verbal (N) features with their descriptions.

**Feature ID**	**Description**
N1	Mean F0 (pitch), representing the speaker's average vocal pitch, averaged across both VF tasks
N2	Mean falling slope of F0, indicating how quickly pitch decreases during falling intonation, averaged across both VF tasks
N3	Mean rising slope of F0, capturing how quickly pitch increases during rising intonation, averaged across both VF tasks
N4	F0 0–2 percentile range averaged across both VF tasks
N5	F0 20th percentile averaged across both VF tasks
N6	F0 median averaged across both VF tasks
N7	F0 80th percentile averaged across both VF tasks
N8	Standard deviation of F0 falling slope, reflecting variability in pitch drop rates, averaged across both VF tasks
N9	Standard deviation of normalized F0, averaged across both VF tasks
N10	Standard deviation of F0 rising slope, averaged across both VF tasks
N11	Mean local jitter, indicating short-term pitch instability (voice roughness), averaged across both VF tasks
N12	Standard deviation of local jitter averaged across both VF tasks
N13	Mean shimmer (dB), reflecting short-term amplitude instability (loudness fluctuations), averaged across both VF tasks
N14	Standard deviation of shimmer (dB), averaged across both VF tasks
N15	Mean phonation perturbation quotient (PPQ), a measure of sustained pitch stability over longer intervals, averaged across both VF tasks
N16	Mean second-order pitch change (delta-delta F0), averaged across both VF tasks
N17	Mean first-order pitch change (delta F0), averaged across both VF tasks
